# Colchicine Improves Clinical Outcomes and Quality of Life in Hidradenitis Suppurativa Patients: A Retrospective Study

**DOI:** 10.3390/jcm10204742

**Published:** 2021-10-15

**Authors:** Aikaterini I. Liakou, Georgios Kontochristopoulos, Efthymia Agiasofitou, Andreas G. Tsantes, Marios Papadakis, Ioannis Marnelakis, Konstantina A. Tsante, Anastasia Kapsiocha, Alexandros Katoulis, Stamatios Gregoriou, Dimitris Rigopoulos

**Affiliations:** 1 First Department of Dermatology-Venereology, Faculty of Medicine, National and Kapodistrian University of Athens, “Andreas Sygros” Hospital for Cutaneous and Venereal Diseases, 16121 Athens, Greece; gkontochris@gmail.com (G.K.); efi.agiasofitou@yahoo.com (E.A.); i_marnerlakis@hotmail.com (I.M.); akapsiocha@hotmail.com (A.K.); stamgreg@yahoo.gr (S.G.); dimitrisrigopoulos54@gmail.com (D.R.); 2 Laboratory of Haematology and Blood Bank Unit, Faculty of Medicine, National and Kapodistrian University of Athens, “Attikon” General University Hospital, 12462 Athens, Greece; andreas.tsantes@yahoo.com (A.G.T.); ktsante@yahoo.com (K.A.T.); 3 Department of Surgery II, University of Witten Herdecke, 58455 Wuppertal, Germany; marios_papadakis@yahoo.gr; 4 Second Department of Dermatology-Venereology, Faculty of Medicine, National and Kapodistrian University of Athens, “Attikon” General University Hospital, 12462 Athens, Greece; alexanderkatoulis@yahoo.co.uk

**Keywords:** colchicine, hidradenitis suppurativa, treatment, quality of life

## Abstract

**Introduction**: Hidradenitis suppurativa (HS) is a chronic inflammatory skin disorder of the follicular epithelium. The aim of the study was to investigate the effectiveness of colchicine on the clinical outcomes of HS patients, and to evaluate wither colchicine as monotherapy or in combination with doxycycline would provide better outcomes. **Methods**: A retrospective study was conducted including 44 patients with established HS, divided into three groups. The first group (n = 15 patients) received colchicine as monotherapy, the second group (n = 14 patients) received colchicine and doxycycline 100 mg/d, while the third group (n = 15 patients) received colchicine and doxycycline 40 mg/d. Disease severity during treatment was assessed at baseline and follow-up, using the Hurley Scoring System and the International Hidradenitis Suppurativa Severity Score System (IHS4). All patients were also asked to complete a Dermatology Life Quality Index (DLQI) questionnaire. These scores were compared among the study groups. **Results**: The DLQI and IHS4 scores significantly improved after treatment with colchicine (*p* < 0.001) in all groups. All colchicine regimes, including the single colchicine regime, colchicine plus doxycycline 100 mg regime, and colchicine plus doxycycline 40 mg regime, resulted in significant improvements in the DLQI and IHS4 scores (*p* < 0.001). Clinical improvement based on DLQI and IHS4 scores was similar in all groups. None of the patients had to discontinue the treatment due to adverse events. **Discussion**: In conclusion, our findings suggest that colchicine may improve clinical severity and quality of life in HS patients, either as monotherapy or in combination with doxycycline, both at antimicrobial (100 mg) and sub-antimicrobial (40 mg) doses.

## 1. Introduction

Hidradenitis suppurativa (HS) is a chronic inflammatory skin disorder of the follicular epithelium, involving primarily the apocrine sweat gland-bearing areas, such as the axillae, inguinal, and anogenital areas. Its prevalence ranges between 1 and 4% in European countries [1,2]. HS has a significant impact on the quality of life of HS patients. A wide variety of medications have been proposed for the treatment of HS, which depends on the morphology, extent, severity, and duration of the disease. Mild HS is usually treated with topical clindamycin, whereas rifampicin combined with oral clindamycin or minocycline are commonly used for stage 1 and 2 HS. Severe cases are treated with cyclosporine, adalimumab, or infliximab and antibiotics. Other HS treatments include oral dapsone, hormone blockers, oral and intralesional prednisone, acitretin, clarithromycin, etc. [3]. Despite the broad armamentarium of drug treatment options, HS management, in both children and adults, may be difficult because of its chronic and recurrent nature. A subset of patients is refractory to standard therapeutic options, making HS treatment even more challenging.

Colchicine is an alkaloid extracted from plants of the lily family, including Colchicum autumnale. Although the exact mechanism of its action is not fully understood, colchicine accumulates in leucocytes and modulates the production of chemokines and prostanoids, decreasing neutrophil degranulation, chemotaxis, and phagocytosis [4]. Its medicinal properties are well-known and there is strong evidence that colchicine is effective in gout and familial Mediterranean fever. Colchicine has been used for a wide spectrum of dermatological disorders, including chronic urticaria, cutaneous vasculitis, actinic keratosis, acne vulgaris, palmoplantar pustulosis, psoriasis, and aphthous stomatitis [5].

It is, therefore, reasonable to hypothesize that colchicine could improve outcomes and quality of life in HS patients. The existing literature on the topic is scarce, since only a few case series have been reported. Van der Zee et al. reported no improvement after treating eight patients with colchicine for up to 4 months [1]. However, the sample was not representative as it was small and only consisted of patients with refractory disease. On the other hand, Armyra et al. treated 20 patients with tetracycline in combination with colchicine and found significant improvements in clinical manifestation and quality of life in all patients [6]. It remains, however, unknown to what extent colchicine contributed to this benefit. It may have been minocycline that was mainly responsible for the changes observed.

The aim of the current study was to investigate the effectiveness of colchicine on the improvement of the clinical symptoms and quality of life of HS patients, and to evaluate whether colchicine as monotherapy or in combination with doxycycline at antimicrobial and sub-antimicrobial doses (100 mg and 40 mg, respectively) would provide better outcomes.

## 2. Materials and Methods

A retrospective study was conducted at the Hospital “Sygrros” from January 2018 to January 2020. Inclusion criteria were patients with established HS who received colhcicine, having a prior 3-month wash-out period and a 3-month follow up. The diagnosis of HS was made according to widely used obligatory and additional criteria [7]. The wash-out period included medications such as systemic steroids, immune suppressive drugs, and non-steroidal anti-inflammatory drugs (NSAIDs). Patients aged < 18 years and patients with known or suspected allergies to doxycycline were excluded from the study. Patients who had received previous treatments were not excluded from the study. Written consent was obtained from all patients. The study was designed according to the principles of the Declaration of Helsinki and was approved by the institutional review board of the hospital.

The patients were divided into three groups. The first group consisted of patients treated with colchicine as monotherapy (1 mg/d). Patients treated with colchicine (1 mg/d) in combination with doxycycline 100 mg/d and patients treated with colchicine and doxycycline 40 mg/d comprised the 2nd and 3rd group, respectively. The decision regarding the treatment regime of the included patients was based on the personal preference of the doctors of the hospital where the study was conducted. All patients were examined at baseline and at the 3-month follow-up visit. Patients were asked to complete a Dermatology Life Quality Index (DLQI) questionnaire. DLQI is a score derived from questions with four alternative responses, ranging from 0 to 30 [8]. Apart from the clinicodemographical data, i.e., age, gender, disease duration, BMI, comorbidities, and family history, disease severity during treatment was also assessed, using the Hurley Scoring System and the International Hidradenitis Suppurativa Severity Score System (IHS4). The latter takes into account the number of nodules (x1), abscesses (x2), and draining tunnels (fistulae/sinuses) (x4), with HS being classified as mild (≤3 points), moderate (4–10 points), or severe (≥11 points) [9].

### Statistical Analysis

Statistical analysis included descriptive statistics of the study population. Normal distribution was determined with histograms, the Shapiro test, and Q-Q plots. Data are presented as means (standard deviations), medians and interquartile ranges (IQR), or percentages when appropriate. The nonparametric Wilcoxon signed rank test was used to compare the scores before and after the colchicine treatment in the study population. The two-sample Wilcoxon rank-sum test and the Kruskal–Wallis test were used for the comparison of differences in score (before and after treatment) among the different colchicine treatment regimes. Multivariable linear regression analysis was also performed in order to further evaluate whether the difference in scores before and after treatment was associated with different colchicine treatment regimes, age, gender, duration of treatment, and Hurley stage. Statistical analysis was carried out using the R software, version 3.5.2. Statistical significance was set at the *p* < 0.05 level.

## 3. Results

A total of 44 patients were included in the study and further analyzed. Of these, 21 were males (47.7%) and 23 females (52.3%). The mean (SD) age was 41.8 ± 14.2 years. The mean (SD) DLQI score and IHS4 score before treatment were 11.1 ± 4.4 and 9.3 ± 3.4, respectively, while the mean (SD) DLQI score and IHS4 score after treatment were 4.5 ± 2.9 and 4.3 ± 2.5, respectively. The mean (SD) duration of the colchicine treatment was 7.3 (8.5) months. Patient characteristics are summarized in Table 1. There was no significant difference in age, gender, IHS4, DLQI, or Hurley scores among the study groups at baseline. Fifteen patients (33%) received colchicine monotherapy, 14 patients (30%) received colchicine plus doxycycline 100 mg treatment, and 15 patients (33%) received colchicine plus doxycycline 40 mg treatment. Patients were not allowed to take NSAIDs for pain management, while antibiotics were not required in any patient. Interventional procedures such as drainages and localized or larger excisions were also not required during treatment. Four patients who received colchicine as monotherapy were Hurley stage I, while 11 patients were stage II. Five patients who received colchicine and doxycycline 100 mg/d were stage I, while seven patients were stage II and two patients were stage III. Lastly, two patients who received colchicine and doxycycline 40 mg/d were stage I, while eight patients were stage II and five patients were stage III. The Hurley stage did not differ among the three study groups (*p* = 0.11). The DLQI and IHS4 scores significantly improved after treatment with colchicine (*p* < 0.001; Table 2). All colchicine regimes, including the single colchicine monotherapy, colchicine plus doxycycline 100 mg regime, and colchicine plus doxycycline 40 mg regime, resulted in significant improvements in the DLQI and IHS4 scores (*p* ≤ 0.001; Table 2). However, the improvement in these scores did not significantly differ among the different colchicine treatment regimens (*p* < 0.05; Table 3). Lastly, multivariable regression analysis further confirmed that the improvement in IHS4 score after colchicine treatment was similar for the different colchicine regimes (coefficient = 0.04, 95% CI: −0.97–1.06), and that it was not associated with age (coefficient = 0.01, 95% CI: −0.04–0.07), sex (coefficient = −1.59, 95% CI: −3.51–0.31), Hurley score (coefficient = 0.24, 95% CI: −1.18–1.68), or duration of treatment (coefficient = 0.04, 95% CI: −0.07–0.15; Table 4). Similarly, multivariable regression analysis further confirmed that the improvement in DLQI score after colchicine treatment was similar for the different colchicine regimes (coefficient = 0.25, 95% CI: −0.99–1.50) and that it was not related to age (coefficient = 0.01, 95% CI: −0.05–0.09), sex (coefficient = −0.26, 95% CI: −2.61–2.08), or Hurley score (coefficient = −1.27, 95% CI: −3.03–0.47), although a longer duration of treatment was found to be related to a greater improvement in the DLQI score (coefficient = 0.44, 95% CI: −0.02–0.30; Table 4). None of the patients had to discontinue the treatment due to adverse events.

## 4. Conclusions

Colchicine is an anti-inflammatory agent that has traditionally been used for the treatment of various dermatological disorders, including chronic urticaria, cutaneous vasculitis, and psoriasis. However, several researchers have raised safety concerns over its side effects and potential toxicity, due to the narrow therapeutic to toxicity window and interindividual variation in drug disposition.

There are only a few studies focusing on the efficacy of colchicine in HS treatment. A prospective Dutch study from van der Zee et al. showed no improvement after colchicine treatment, with the patients reporting frequent side effects, such as nausea and diarrhea [1]. However, this case series consisted of only eight patients with disease refractory to several other treatments, i.e., oral antibiotics, oral contraceptives, isotretinoin, resorcinol, and surgical treatments. It is therefore questionable whether the findings can be generalized to all HS patients. Additionally, HS is a chronic disorder and only two of the eight patients (25%) were treated for 4 months. Even though the therapeutic efficacy of colchicine is evident within 1 week of therapy [6], a longer treatment regimen is required in disorders such as Behcet’s disease, where colchicine must be used for years [4]. The most common reason for dropping out from the Dutch study was a lack of efficacy. Only one patient stopped the treatment because of side effects. In our study, the mean treatment duration was 8 months and a longer duration of treatment was found to be related to a greater improvement in DLQI score. Thirdly, as the authors themselves reported, the lack of efficacy of colchicine observed may have been attributed to initial underdosing: the authors administered 1 mg of colchicine daily, whereas two patients received 1.5 mg daily after the first month. It is unknown whether a higher starting dose followed by a maintenance dosage would have improved the efficacy. However, in our opinion, 0.5 mg colchicine administered twice daily (1 mg/d) is effective from a long-term perspective. As mentioned above, colchicine has a narrow therapeutic to toxicity window, the therapeutic plasma levels being achieved with 1–2 mg colchicine per day [4].

Armyra et al. combined the anti-inflammatory action of both colchicine and minocycline in a prospective study of 20 patients [6]. All patients were treated with 100 mg oral minocycline in combination with 0.5 mg colchicine administered twice per day for 6 months. The maintenance regimen consisted of 0.5 mg colchicine administered orally twice per day for 3 months. Efficacy was evaluated by means of a physician’s global assessment (PGA) scale at 3-month intervals. The authors reported a significant improvement in disease manifestation. Minocycline is, however, not common in HS treatment, probably because it is associated with a higher risk of hypersensitivity syndrome and drug-induced lupus [10]. Doxycycline is generally preferred, as in our study, because it can be taken with food [10].

DLQI is the most widely used tool to assess quality of life in HS patients. Surgical interventions and photodynamic therapy have been shown to reduce the DLQI score by more than 80%, whereas the aforementioned combination of minocycline with colchicine decreases the DLQI by 60% [11]. We found a mean overall DLQI score of 11, before treatment. DLQI scores greater than 10 indicate that the skin disease has a very strong effect on the patient’s life. Such scores are generally considered to be strong supportive evidence for the need for active intervention [12]. In our study, after treatment, the overall score decreased to <5, i.e., a score showing a minor effect of the disease on the patient’s life [11]. There was no difference between groups, indicating that colchicine can improve quality of life, both as monotherapy and in combination with doxycycline.

Doxycycline has been employed successfully in the treatment of HS because of its antimicrobial, anti-inflammatory, and immunomodulating properties. Interestingly, there was no difference between patients receiving 100 mg doxycycline and 40 mg doxycycline (Oracea^®^), combined with colchicine. Oracea^®^ contains doxycycline 30 mg immediate-release and 10 mg delayed-release, providing a sub-antimicrobial dose of doxycycline. It has been proven to be equally as effective as 100 mg doxycycline in patients with moderate to severe rosacea and patients with moderate and severe acne [13,14]. Not unexpectedly, its tolerability profile appears to be more favorable than that of doxycycline 100 mg. However, the problem of inducing resistant intestinal bacteria cannot be ruled out with the long-term use of sub-antimicrobial dosages of doxycycline [13]. Pallasch reports that two daily doses of 20 mg doxycycline produce blood levels of 0.79 micrograms/mL, and doxycycline is effective in the management of infectious diseases at serum dose levels >0.04 micrograms per ml and life-saving (infections caused by vancomycin-resistant enterococci and staphylococci) at blood levels of 0.06 to 0.25 micrograms/mL [15]. Oracea^®^ is often combined with topical metronidazole 1% gel [13]. We administered 40 mg capsules, once daily on an empty stomach, with efficacy comparable to colchicine−100 mg doxycline and colchicine alone.

There are some limitations of the study that must be addressed. First, flare-ups during treatment were not recorded. This could have confounded our conclusions regarding the efficacy of each treatment regime. Second, this was a small retrospective study without a placebo control group. Evaluation of such a group would be valuable, since a beneficial effect of stopping potentially worsening drugs such as systemic steroids, immune suppressive drugs, and NSAIDs cannot be ruled out. This should be further assessed in a future study along with the evaluation of flare-ups during treatment. Third, the retrospective nature of the study and therefore the suboptimal group assignment is a certain limitation of the study. Since this was a retrospective study, we aimed to simply evaluate and compare the already collected data from the patients who received one of the three treatment regimes. However, we performed logistic regression analysis to adjust our results for any confounding factors due to the different baseline characteristics of the patients.

In conclusion, our findings suggest that colchicine may improve clinical severity and quality of life in HS patients, either as monotherapy or in combination with doxycycline, both at antimicrobial (100 mg) and sub-antimicrobial (40 mg) doses.

## Figures and Tables

**Table 1:** Characteristics of the study group.

	Study Group (n = 44)
Age, years	41.6 ± 14.2; 41.8 (33.0–51.0)
Gender (males)	21 (47.7)
Duration of colchicine treatment (months)	7.3 ± 8.5; 3.0 (2.0–9.0)
Colchicine treatment	
Single colchicine	15 (34.1)
Colchicine + Doxycycline 100 mg	14 (31.8)
Colchicine + Doxycycline 40 mg	15 (34.1)
Hurley stage	
Stage I	11 (25.0)
Stage II	26 (59.1)
Stage III	7 (15.9)
DLQI before treatment	11.1 ± 4.4; 12.0 (8.0–14.0)
IHS4 before treatment	9.3 ± 3.4; 10.0 (8.0–12.0)
DLQI after treatment	4.5 ± 2.9; 4.0 (2.0–6.0)
IHS4 after treatment	4.3 ± 2.5; 4.0 (2.0–6.0)

Data are shown as means ± SD, medians and interquartile ranges (IQR), or as n (%) where appropriate.

**Table 2:** Comparison of scores before and after the colchicine treatment regimes.

Treatment	IHS4			DLQI		
	Before Treatment	After Treatment	*p* Value	Before Treatment	After Treatment	*p* Value
Overall (n = 44)	9.3 ± 3.4; 10.0 (8.0–12.0)	4.3 ± 2.5; 4.0 (2.0–6.0)	**<0.001**	11.1 ± 4.4; 12.0 (8.0–14.0)	4.5 ± 2.9; 4.0 (2.0–6.0)	**<0.001**
Single colchicine (n = 15)	7.7 ± 3.3; 8.0 (6.0–10.0)	2.8 ± 1.4; 2.0 (2.0–4.0)	**<0.001**	9.8 ± 4.2; 10.0 (8.0–14.0)	3.4 ± 2.3; 3.0 (2.0–5.0)	**<0.001**
Colchicine + Doxycycline 100 mg (n = 14)	11.1 ± 2.9; 12.0 (10.0–12.0)	5.8 ± 1.9; 6.0 (4.0–8.0)	**<0.001**	13.0 ± 3.9; 13.0 (12.0–16.0)	6.3 ± 3.2; 6.0 (4.0–10.0)	**0.001**
Colchicine + Doxycycline 40 mg (n = 15)	9.9 ± 3.6; 10.0 (8.0–12.0)	4.5 ± 2.5; 4.0 (2.0–6.0)	**<0.001**	11.6 ± 4.8; 12.0 (10.0–12.0)	4.5 ± 2.2; 4.0 (4.0–6.0)	**<0.001**

The Wilcoxon signed rank test was used for comparison before and after treatment.

**Table 3:** Comparison of score improvements (before and after treatment) among the different colchicine treatment regimes.

	Single Colchicine (A = 15)	Colchicine + Doxycycline 100 mg (B = 14)	Colchicine + Doxycycline 40 mg (C = 15)	Overall Comparison	A vs. B; A vs. C; B vs. C
DLQI improvement	6.3 ± 3.3; 6.0 (5.0–10.0)	6.6 ± 3.8; 6.0 (4.0–10.0)	7.0 ± 4.1; 7.0 (4.0–9.0)	*p* = 0.52	*p* = 0.92; *p* = 0.78; *p* = 0.82
IHS4 improvement	4.9 ± 3.0; 4.0 (4.0–6.0)	5.2 ± 1.6; 5.0 (4.0–6.0)	5.4 ± 3.6; 4.0 (4.0–6.0)	*p* = 0.73	*p* = 0.43; *p* = 0.96; *p* = 0.56

Data are presented as means ± SD, medians and interquartile range (IQR). The two-sample Wilcoxon rank-sum (Mann–Whitney) test was used for the 2 × 2 comparisons between the groups, while the Kruskal–Wallis test was used for the overall comparison among the 3 groups.

**Table 4:** Results of multivariable linear regression analysis for differences in IHS4 and DLQI scores as dependent variables and colchicine regime, sex, age, duration of treatment, and Hurley stage as independent variables.

	IHS4			DLQI		
	Coefficient	95% CI	*p* Value	Coefficient	95% CI	*p* Value
Sex	−1.59	−3.51–0.31	0.10	−0.26	−2.61–2.08	0.82
Age	0.01	−0.04–0.07	0.70	0.01	−0.05–0.09	0.43
Hurley score	0.24	−1.18–1.68	0.72	−1.27	−3.03–0.47	0.14
Colchicine regime	0.04	−0.97–1.06	0.92	0.25	−0.99–1.50	0.68
Duration of treatment	0.04	−0.07–0.15	0.44	0.16	0.02–0.30	**0.024**

## Data Availability

Data are available on request from the corresponding authors.
